# Collaboration between traditional health practitioners and biomedical health practitioners: Scoping review

**DOI:** 10.4102/phcfm.v16i1.4430

**Published:** 2024-07-31

**Authors:** Ngcwalisa A. Jama, Anam Nyembezi, Sekgameetse Ngcobo, Uta Lehmann

**Affiliations:** 1 School of Public Health, Faculty of Community and Health Sciences, University of the Western Cape, Cape Town, South Africa; 2 Health Systems Research Unit, South African Medical Research Council, Cape Town, South Africa

**Keywords:** biomedical health practitioners, traditional health practitioners, traditional healers, collaboration, integration, bidirectional collaboration, pluralistic healthcare, mental health

## Abstract

**Background:**

Collaboration between traditional health practitioners (THPs) and biomedical health practitioners (BHPs) is highly recommended in catering for pluralistic healthcare users. Little is known about bidirectional collaborations at healthcare service provision level.

**Aim:**

To map global evidence on collaboration attempts between THPs and BHPs between January 1978 and August 2023.

**Method:**

We followed the Arksey and O’Malley framework in conducting this scoping review. Two reviewers independently screened articles for eligibility. A descriptive numerical and content analysis was performed on ATLAS.ti 22. A narrative summary of the findings was reported using the PRISMAScR guideline.

**Results:**

Of the 8404 screened studies, 10 studies from 12 articles were included in the final review. Studies came from America (*n* = 5), Africa (*n* = 2), China (*n* = 2) and New Zealand (*n* = 1). Eight studies reported case studies of bidirectional collaboration programmes, while two studies reported on experimental research. All collaborations occurred within biomedical healthcare facilities. Collaboration often entailed activities such as relationship building, training of all practitioners, coordinated meetings, cross-referrals, treatment plan discussions and joint health promotion activities.

**Conclusion:**

This study confirmed that practitioner-level collaborations within healthcare are few and sparse. More work is needed to move policy on integration of the two systems into implementation. There is a need to conduct more research and document emerging collaborations.

**Contribution:**

This research illuminates the contextual challenges associated with sustaining collaborations. The data would be important in informing areas that need strengthening in the work towards integration of THPs and BHPs.

## Introduction

Healthcare seekers across the world utilise multiple systems of care.^1,2,3,4^ Traditional health practitioners (THPs) are often consulted or even preferred, alongside mainstream, allopathic practitioners, because they are potentially more accessible, available, affordable and culturally acceptable for holistic care.^5,6,7,8,9,10^ Indeed, a recent World Health Organization (WHO) global survey found that in most countries (93 of 133), THP services are highly utilised.^11^ The WHO defines THPs as practitioners who use indigenous or native approaches, knowledge, culture and spiritual therapies, as well as beliefs that incorporate plant-, animal- and/or mineral-based medicines to maintain well-being.^12^

This definition provided by WHO is often used to encompass all traditional healers, and other alternative and complementary healers. As such, in the global literature, there is usually a conflation of traditional healers with complementary and alternative medicine (CAM). The latter often refers to traditional medicine or healing practiced outside of its origin and traditional culture, thus less culture-specific.^13^ Furthermore, CAM is distinguished by being far advanced in policy development and regulations.^14,15,16,17,18^ On the other hand, traditional healing practices are localised, deeply rooted in spirituality and still marginalised as a healthcare system in most parts of the world, when compared to CAM.^19^ For this study, the focus is on traditional healers and any reference to THP only refers to indigenous traditional healers and not CAM.

Over the past few decades, the WHO has sought to support countries in harnessing the contribution of THPs in the healthcare system. For example, the WHO’s Traditional Medicine Strategy released in 2002 and updated in 2013 highlights the context, which can foster a more integrated health system, where THPs are fully part of the health system.^20,21^ Despite the important progress that has been made in this area, there are still significant limitations.^11,22,23,24,25^ As early as 1978, the WHO stressed that partnership between THPs and biomedical health practitioners (BHPs) would be key in the process of integration.^12,20^ Since then, increased attention has been placed on how to foster practitioner-level collaborations.^11,26,27,28^ This work has involved investing time and resources into studying traditional medicines and herbs, institutionalisation of THPs and patients views on such collaborations, as well as the establishment of healthcare centres for THPs and BHPs collaborations in well-resourced parts of the world.^15,16,17^

In Africa, disease outbreaks such as human immunodeficiency virus (HIV) have led to increased literature on collaborative partnerships between THPs and BHPs.^29,30^ King and Homsy^29^ give a summary of these attempts in the area of HIV in their review. Sima and colleagues^31^ provide an example of recent attempts of THPs and BHPs collaboration from TB cases in Ethiopia. In these collaborations, THPs are trained to refer clients to BHPs, as such they have been criticised for being one-sided, leaving practitioners and policymakers with little to learn from these attempts.^30,32,33,34^

For collaboration to be reciprocal, it requires a process where THPs and BHPs form partnerships which, at the very least, constitute an act of client referral between two practitioners.^12,20,22^ This definition does not provide a clear indicator of how these referrals should occur and does not mention other components that should be present in a collaboration. D’Amour et al.^35^ maintain that collaboration extends beyond forming partnerships, and involves mutual trust and respect, having a set of common goals, sharing of values, responsibilities, power and decision-making, as well as interdependency in addressing patients’ needs. This interdependency facilitates co-treatment or bidirectional referral of patients between practitioners.

Similar to D’Amour and colleagues, Kraus^36^ describes collaboration in healthcare as:

[*A*] cooperative venture based on shared power and authority. It is non-hierarchical in nature. It assumes power based on knowledge or expertise as opposed to power based on role or function. (p. 12)

Therefore, genuine collaboration necessitates a two-sided effort whereby the healing methods of one are brought to the fore and the most effective one is chosen to cure the patient’s identified problem at that time.^37^

As seen from these definitions, collaboration must constitute reciprocity, and any one-way partnership cannot be referred to as collaboration. Recent studies by Ampomah et al.,^38^ Mutale,^39^ and Baheretibeb, Wondimagegn and Law^40^ point to the continued paucity of collaboration reports between THPs and BHPs. These scholars repeat the same barriers that have been highlighted in most studies in this area. These mainly include philosophical difference in how the practitioners from the two systems view health and approach treatment, countries’ resources and political willingness to incorporate THPs into the health systems, and administrative barriers in ensuring partnerships are formed and sustained between THPs and BHPs. Despite these challenges, THPs and BHPs are encouraged to formulate functional collaborations as the service users continue to use them both, concurrently.^39^

As stated by Kayombo et al.^41^ and others earlier, information on the availability of collaboration for patient care is missing. In response to this, this study sought to scope and understand whether collaborations of this nature potentially exist; if so, which salient factors are associated with the success (or lack of) of these collaborations. Understanding these factors is useful for the conceptualisation of pragmatic collaboration attempts, as well as the development of collaboration frameworks, which will inform future policy and practice. We therefore mapped literature on collaborations between THPs and BHPs globally, across all health conditions and based on the definition of collaboration described earlier.

## Methods

### Design

A scoping review method was conducted to investigate the availability and type of evidence that is available regarding collaborations between THPs and BHPs, globally. We applied the methodological framework by Arksey and O’Malley^42^ and the 2017 recommendations of the Joanna Briggs Institute (JBI)^43^ in identifying, selecting and extracting data for this review. We then analysed data on ATLAS.ti 22. A detailed scoping review protocol outlining the methodological techniques we followed was published in BMJ Open.^44^ Any deviations to what was proposed in the protocol have been reported in Appendix 1. The review report follows the PRISMA-ScR (Preferred Reporting Items for Systematic Reviews and Meta-analysis: Extension for Scoping Review) guidelines.^45^

### Identification of relevant literature

To identify relevant literature, eight databases were used, such as EMBASE, PubMed, MEDLINE, APA PsycArticles, Cumulative Index of Nursing and Allied Health (CINAHL) Plus, Academic Search Complete, Latin American and Caribbean Health Science Literature (LILACS) and Scopus from 1978 to March 2020. This search was updated in November 2022 in the three databases (MEDLINE; CINAHL plus; Academic Search Complete), which had the most relevant records during the first search. A second update of the search was performed in August 2023 in the same three databases. Literature search was also conducted using free-text words on bibliographical search engines such as Google Scholar and Academia.edu., national health departments, the WHO repository, and the Open Access Theses and Dissertations library. We found additional studies from the reference lists of similar studies. We used the JBI (2017)^43^ PCC mnemonic (population, concept and context) to define the research question to guide the search strategy (Appendix 2). Following are the steps we undertook to refine search strategy.

#### The search strategy

A preliminary search was conducted on Google and Google Scholar to explore terms that are used to refer to traditional healers, as these terms differ in the global and local literature. This assisted in the choice of the terms that were used in the review. For instance, we used the term ‘alternative healers’ to allow selection of all studies that use this broad term. Briefly, the search strategy included a set of keywords on the collaboration of THPs and BHPs, with the help of a library specialist for electronic bibliographic search. The reviewers’ initial feedback on the published scoping protocol also helped in refining our search strategy. The initial and updated search strategy is shown in Appendix 3. Considering the variety of THPs worldwide, inclusion criteria for the search entailed THPs who are classified as traditional healers, diviners and herbalists, alternative healers, native healers, aboriginal healers, indigenous healers, traditional Chinese healers, traditional native healers, Shammas, with the exclusion of faith or spiritual healers, traditional birth attendants, herbalists and complementary medical practitioners.

We also included studies that focused on all health conditions, those published in English from 1978 to March 2020 as well as 1978 to August 2023. The detailed search inclusion and exclusion criteria are outlined in the PCC framework in Appendix 2.

### Study selection

Once records had been organised and deduplicated on the EndNote X platform, two reviewers (N.A.J. and S.N.) screened the articles for selection on Rayyan QCRI Systematic Reviews Web Application.^46^ Before commencing with screening, the two screeners met to discuss the process, trial the screening tool and agree on screening instructions and objectives. Title and abstract screenings were conducted in duplicate. The same screening method was applied for full-text screening. Any conflicts generated through the screening stages between the two reviewers were discussed until consensus was reached.

### Data extraction

Data extraction of selected full-text articles was conducted on the REDCAP,^47^ using a tool to record the following: article characteristics (first author’s name, year, region and country), population characteristics (sample size and approach), intervention characteristics (nature of the intervention, intervention components, intervention duration, and reasons for ending collaboration, context of the intervention, key barriers and enablers to collaboration) and key findings. The tool can be found in Online Appendix 1. The authors N.A.J. and S.N. conducted double extraction on the first seven articles. Findings from the double collection of data were compared and validated, and there were no major differences found. After this, N.A.J. completed the extraction process. Once extraction was completed, the data were exported into an Excel sheet for cleaning and organising. Finally, the data were analysed.

### Synthesis of results

A descriptive account of included studies was prepared to present the nature and context of the collaborations. Basic numerical analysis was also used to understand geographic distribution of the studies, the type of studies included and other relevant demographics. The first author analysed the data on ATLAS.ti 22 and shared it with all authors for review and discussion.

### Patient and public involvement

No patients or members of the public were involved in the design of this scoping review.

## Review findings

### Selection of sources of evidence

**FIGURE 1:**
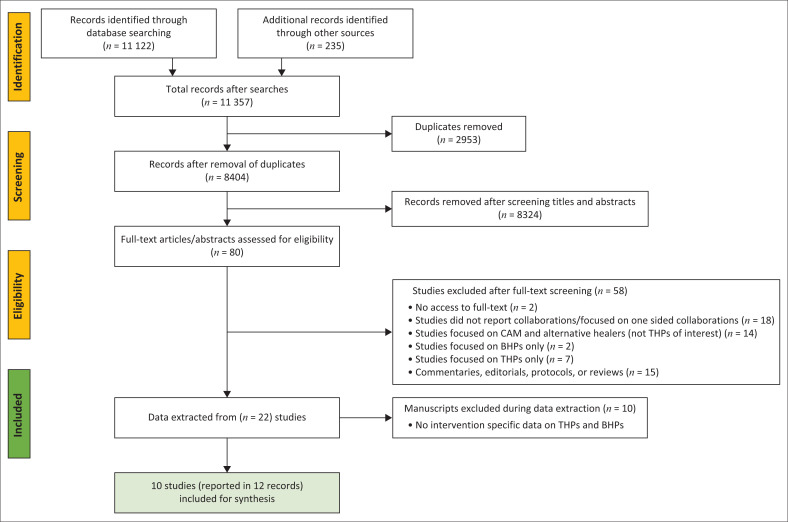
Preferred Reporting Items for Systematic Reviews and Meta-Analyses flow diagram.^45^

A total 11 357 citations were identified (see Figure 1). Results were exported to Endnote X8 and 2953 duplicates were deleted. A total of 8404 articles were exported to Rayyan^47^ for screening. Of the 8404 remaining articles, 8324 articles were removed upon screening article titles and abstracts based on the eligibility criteria (see Appendix 2). While we were able to eliminate most of the records that did not meet our eligibility criteria during the search phase, an overwhelming number of unrelated records made it into our search because they included data on CAM practitioners, which are sometimes termed THPs. Furthermore, most records were based on opinions of collaboration and not the actual practice of collaboration, thus were excluded.

Full-text screening was conducted on 80 articles and 58 of these articles did not meet the inclusion criteria. The reasons for excluding the 58 articles are mentioned in the PRISMA flow diagram (Figure 1). It is worth mentioning that it is uncommon to exclude studies for not meeting the ‘population of interest’ criteria at full-text screening stage, but this occurred because it was impossible to exclude these studies at abstract and title screening because of the unclear distinction of THPs at this stage. As stated in the background, majority of literature in this area use the WHO’s definition of THPs, which also encompasses CAMs. Twenty-two studies that were considered eligible were analysed for this scoping review. Ten more studies were excluded after analysis, as we could not distinguish specific data on collaboration between THPs and BHPs. The final number of studies that met our inclusion criteria was 10, and these were described in 12 articles. Studies by Gureje et al.^48^ and Joe et al.^49^ were described in two articles, and articles with richer and detailed reporting were chosen for synthesis. The authors did not perform quality appraisal of the selected studies; therefore, no studies were excluded on the basis of quality.

### Characteristics of sources of evidence

**FIGURE 2:**
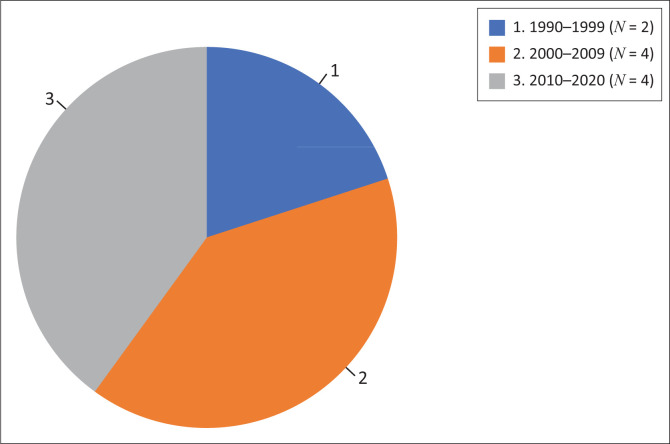
Studies published by year (*N* = 10).

The studies presented two kinds of evidence. The type of evidence included here *were case study reports*^49,50,51,52,53,54,55,56^ of established collaboration (*n* = 8) and *experimental research interventions*^48,57^ on THP/BHP collaboration (*n* = 2). Research methods used on the research intervention studies were quantitative randomised trial^48^ and pre-post evaluation quantitative study.^57^ The years the studies were published are outlined in Figure 2. We found two (*n* = 2) studies from 1990 to 1999, four (*n* = 4) studies in 2000–2009 and four (*n* = 4) studies from 2010 to 2020.

## Results of sources of evidence

The description of studies, intervention characteristics and findings of studies have been added in Online Appendix 2.

**FIGURE 3:**
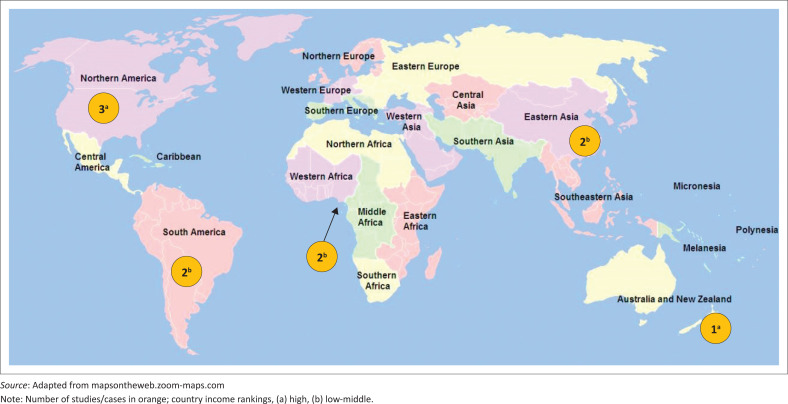
Number of included studies by world regions and subregions.

As seen in Figure 3, research on collaboration between THPs and BHPs came from Southern America (*n* = 2),^50,51^ Northern America,^49,54,55^ Africa (*n* = 2),^48,57^ China (*n* = 2)^53,56^ and New Zealand (*n* = 1).^52^ Out of the 10 included studies, 4 were conducted in high-income countries (HICs)^49,52,54,55^ and 6 in low- and middle-income countries (LMICs).^48,50,51,53,56,57^

### Medical condition for which collaboration was initiated

Half (5/10) of the collaborations found in this review focused on the treatment and management of mental illness conditions.^48,51,52,54,55^ These studies were from the United States (*n* = 3),^51,54,55^ Africa (*n* = 1)^48^ and New Zealand (*n* = 1).^52^ Kaboru et al.^57^ investigated collaboration within the context of HIV and acquired immunodeficiency syndrome (AIDS) management. In three studies,^51,53,56^ collaborating practitioners saw clients for acute to mild ailments such as wound care, diabetes, physical trauma, pain management, etc. The medical conditions treated were not specified in the study by Joe et al.^49^

### Nature of collaboration

Based on the definition of collaboration and description of THPs adopted in this study, we present collaborations that occurred in both clinical/real-world setting (*n* = 8)^49,50,51,52,53,54,56,58^ and those that were formulated as part of a research intervention (*n* = 2).^48,57^ For both settings, we present findings related to the structure of the intervention (involved personnel, collaboration setting and duration), collaboration processes (factors related to activities, decision-making and power dynamics within the collaborations) and challenges to sustainability.

#### Collaboration structure (setting, support, personnel and duration)

In four studies,^49,52,55,56^ collaboration took place in a dedicated unit within a biomedical hospital setting. In these settings, collaboration was enabled by hiring THPs to join a biomedical team for specific programmes run by specific units within the hospitals and for specific durations. In the hospital settings, practitioners were co-located either within the same unit, but different offices or had a department completely belonged to them. In other four studies, collaborations happened at community healthcare centres (CHCs).^50,51,53,54^ Two of the four CHCs were built for the sole purpose of having THPs and BHPs working together in providing culturally appropriate services.^51,54^ The development of the two CHCs was enabled by financial support from various sources such as sponsors or the health departments, which also helped with the continuity of these centres. The integrated care CHC presented in the report by Bouchard^51^ closed after 2 years because of the lack of support from the government. The other two CHCs existed long before the introduction of THPs, and collaboration is still ongoing in these centres.^50,53^ In the two remaining studies, collaboration occurred as part of research and included one pre-post intervention study^57^ and one randomised control trial intervention study.^48^ Both studies were located in Africa and lasted for up to 12 months. Collaborations, whether occurring in clinical or research setting and despite being in HICs or LMICs, were in rural settings and aimed at indigenous populations.

All collaborations constituted of THPs and BHPs. However, in the study by Joe et al.,^49^ the role of THPs was taken by practitioners with dual training as BHPs and THPs. THPs from Africa were all referred to as African THPs. Studies from Northern America referred to THPs as Quichua traditional healers in one study^51^ and Navajo native healers in another study.^49^ In the study from New Zealand, THPs were referred to as Māori healers^52^ and traditional Chinese healers in China. Only 4 of 10 studies mentioned the composition of the collaborating practitioners.^49,51,53,57^ According to Kaboru et al.,^57^ there were 28 THPs and 19 BHPs that formed part of the collaboration team. Bouchard^51^ stated that 2 THPs and 10 BHPs, as well as 2 media graduates were involved in the collaboration. Joe et al.^49^ mentioned three THPs with dual training. Kushner and Yu^53^ mentioned 19 THPs and the number of BHPs was not stated.

#### Collaboration processes

**FIGURE 4:**
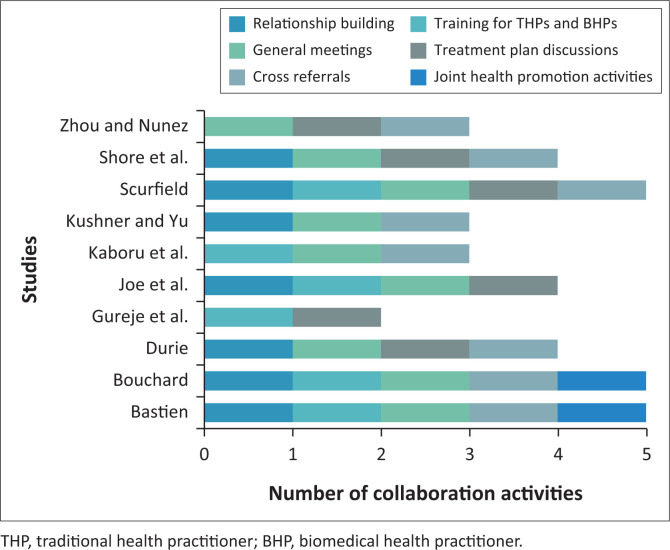
Main collaboration activities identified across studies.

Content analysis revealed six main collaboration activities across all studies. These activities are tabulated as shown in Figure 4.

Figure 4 shows that activities related to conducting general meetings, followed by the practice of cross-referrals, and attempts at relationship building between practitioners were common. The practice of planning treatment plans for clients and having joint trainings and health promotion activities for THPs and BHPs was found in some studies. These are discussed further in the following sections.

**Relationship building:** For most studies, collaboration started as the results of institutional formalised processes, such as the formal recruitment of THPs into biomedical spaces and building relationships with the THPs.^49,52,54,55^ In the study conducted by Durie,^52^ the inclusion of THPs into the CHCs involved signing of contracts between the District Health Boards and Māori traditional healers, as well as developments of protocols, which reflect Māori values of communication, within the CHCs. In Kushner and Yu^53^, as well as Zhou and Nunez,^56^ THPs were already located in hospitals or CHCs, and only interpersonal relationships had to be enforced.

**Training of traditional health practitioners and biomedical health practitioners:** Once the relationship was built between practitioners, a series of training activities took place. The duration of the training differed across the studies, ranging between 2 days and 2 weeks. The training mainly focused on the orientation of the practitioners on the different treatment approaches and facilitating information exchange sessions. As part of the training, practitioners joined treatment sessions of one another as a way of learning how treatment is administered by the peers. This was reported in studies by Bastein,^50^ Scurfield^54^ and Joe et al.^49^

**Cross-referrals, general meetings, treatment plan discussions, joint health promotion activities:** Other collaboration activities included cross-referrals, meetings, discussions of treatment plans and joint health promotion activities. In 9 of the 10 studies, practitioners practiced independently of each other, even though they were co-located or part of the same intervention programme.^49,50,51,52,53,54,55,56,57^ Therefore, collaboration happened through referrals. These referrals were ad hoc and either practitioners or clients facilitated. In their study, Kushner and Yu^53^ stated that 20% and 30% of the patients seen by TH practitioners were referred directly by BHP. This was reciprocated by THPs who referred the same amount of their patients to BHPs. In the study by Joe et al.,^49^ over 50% of their patients were self-referrals.

The process of referral in one health facility is summed by Bouchard^51^ as follows:

The patient would come to the clinic and ask to see either the yachactaita (Quichua traditional healer) or the Western trained doctor. The chosen practitioner would examine the patient, make a diagnosis, and propose a treatment plan. (p. 85)

Here, patients would see the other practitioner if not satisfied with treatment. In all nine studies practicing cross-referrals, it was part of practitioner’s responsibilities to follow-up on referrals made and document the referrals on their individual client charts. In six studies, practitioners had treatment plan discussions.^48,49,52,54,55,56^ The practitioners teamed up to also work on health promotion services, such as childhood vaccination drives.^50,51^

#### Threats to sustainability of collaborations

**Short time frame:** Three of the 10 included studies^48,54,57^ had a predetermined collaboration time frame, and as such there was no requirement to sustain collaboration beyond the agreed time frame. For instance, the collaboration programme reported in the study by Scurfield^54^ was set to take place for 11 weeks. In the study by Gureje et al.,^48^ the authors mentioned that the collaboration was only set to take place for 3 months, and in the study by Kaboru et al.,^57^ collaboration was set for not more than 12 months.

**A lack of resources:** Regardless of whether collaborations were set for a limited time frame or were set to continue, challenges with sustaining collaborations were mentioned in almost all studies. The challenges ranged from relational, administrative to political interference. In studies where collaboration was meant to be ongoing, these were challenges that impacted the continuity of the collaboration, as observed in the study by Bouchard.^51^ Here, the lack of resources and political interference led to the ending of collaboration programme. According to Bouchard:^51^

[*L*]eftist political organization with which Jambihuasi had been collaborating tried to take control of the health center. They began campaigning in the surrounding Quichua communities, with the argument that the center was the property of the true poor Quichua, and convinced a number of people that they should take over Jambihuasi by any means necessary. (p. 87)

Kushner and Yu^53^ argued that THPs’ experience of a lack of respect from the BHPs’ counterparts was a major barrier to smooth collaboration.

**Philosophical differences and adminis tration issues:** Administrative challenges by both the BHPs and THPs were observed in the documentation and categorisation of illnesses, for example, with the use of ICD-10 codes in three studies. In a focus group discussion, one BHP in the study by Joe et al.^49^ commented:

[*S*]pirituality not part of the language used in the medical arena. The medical training did not include a course on how to treat patients needing this kind of service, nor did it teach about assessments or a laboratory test that indicated a patient was low on his Native medicine. (BHP comment during focus group discussion in Joe et al.; p. 34)^49^

Zhuo and Nunez^56^ state that administrative challenges observed in their settings were linked to philosophical differences, which hindered treatment plan discussions. One interviewed BHP stated:

Traditional Chinese medicine diagnosis uses inspection, listening and smelling examination, inquiry, and palpation. These methods are far different from ours. Sometimes we suspect how accurate these methods are. (THP interview extract in Zhou & Nunes; p. 244)^56^

In the hospital in China, Zhou and Nunes^56^ mentioned that treatment was administered separately, with little room for discussing patient progress even when referrals had been made. Another THP in this study said:

[During consultation] usually they [BHP] do not ask many questions, and we do not talk that much. We all are very busy. As long as we can treat the patient, that is all right. We all are too busy to actually sit down and to have a deep conversation. (THP interview extract in Zhou & Nunes; p. 243)^56^

Findings from the study by Durie^52^ mentioned challenges with fitting culture-based illnesses into the diagnostic manual (DSM IV) and this led to tension between THPs and BHPs in a case from New Zealand. Similarly, the introduction of indigenous practices led to extra-administrative demands on health facilities.

## Discussion

This scoping review, covering the past five decades, demonstrates that there is a dearth of evidence on collaboration practices between THPs and BHPs globally. With only 10 studies reporting THP and BHP bidirectional collaboration, our study shows that while literature on collaboration between THPs and BHPs exists, as seen from the number of studies screened and later excluded in this review, only a fraction of this literature reports actual reciprocal collaborations between the two healthcare systems. This study was able to show the availability, as well as describe the structure, dynamics and overall contributing factors to success or lack of success of collaborations, globally.

The available publications on collaboration were sparse, with only four studies published in the last decade in this area. However, we caution that it is likely for collaboration endeavours to go undocumented, as we found that in publication information of two studies by Bouchard^51^ and Durie.^52^ The reported interventions were implemented in 1990 and 1980, but only published in 2009. In the two studies reported from Africa, neither reported real-life collaboration programmes, but rather experimental research studies. Likewise, these collaborations had a short time frame with no plan of sustaining the programmes. Street et al.^59^ mentioned how these research efforts do not consider the scale up of intervention programmes. As the studies included were research projects, occurring outside of the daily functioning of the health facilities, making them difficult to imagine as a day-to-day clinical practice, it is difficult to make implementation recommendations from these studies. This finding indicates that more studies are needed to understand reasons for continued lack of collaboration programmes in this region. The evidence from other countries was just as limited, with only five studies presenting collaboration cases in America, two studies in Asia and one study in New Zealand.

It was surprising to see little literature reporting collaboration in countries such as China. While we excluded records on the CAM practitioners, we still expected to find data on Chinese THPs considering that the country has taken huge strides in integrating traditional Chinese healers into the health system. According to Canaway^15^ and Pinkoane,^60^ the integration of THPs in China has mainly happened at the governance or constitutional level, as seen with the transformation of the education to include both systems. The focus has also been in the integration of THP medicine, compared to the formation of reciprocal collaborations between THPs and BHPs in this setting. Park and Canaway^28^ state that Chinese practitioners are still trying to forge partnerships. Zhou and Nunes^56^ and recently Boum^61^ confirm that there are interpersonal and training differences that make it difficult for the two practitioners to collaborate, despite the seemingly enabling environment.

Out of all studies analysed here, THP-BHP collaboration was mostly found for mental health services. Our findings revealed that there seems to be a focus of collaboration towards severe disorders within mental health, such as psychosis. Mental health conventional practitioners have long recognised the potential of THPs within the space of mental health management.^13,62,63,64,65^ Our evidence shows that, while THPs and BHPs seem to be doing better in initiating collaborations within mental health, more work needs to be performed to strengthen the administration within these collaborations. For example, we found that THPs complained about inability to record their diagnosis on client files, as spiritual illnesses are not accommodated. The current Diagnostic and Statistical Manual of Mental Disorders (DSM-5-TR)^66^ tool does make mention of culture-bound illnesses; however, it is possible that this tool places different spiritual related illness under one diagnosis, mostly referred to as culture-bound syndrome.

This review allowed for an understanding of collaboration structures, processes and collaboration dynamics, as well as an overview of challenges with forming and sustaining collaboration. Martín-Rodríguez et al.^67^ write that for collaboration to efficiently happen, changes need to happen at systematic, organisational and interactional levels. Collaboration especially in HICs was mostly mandated. This mandate to create person-centred integrated healthcare came from the health ministries and the healthcare seekers. This was followed by infrastructural changes, availability of leadership support and financial resources.

Durie,^52^ who reported a case study with least reported challenges, described how collaboration rather started at policy level, where there was a political shift to embrace indigenous ways of healing and foster culture-sensitive healing in New Zealand. In their study, integration filtered down to university level where health sciences students were taught about Maori traditional healing methods, which enabled them to appreciate working with THPs when government enforced collaboration.

When it comes to the organisation of collaborations, reports from all studies showed that THPs had to leave their community-based practices to be placed at a hospital healthcare centre or a research site, where they would work. Shortell et al.^68^ and Coddington, Fischer and Moore^69^ warn that this acute care approach is a cultural barrier to collaboration and contradicts the concept of population-based healthcare delivery. It also corroborates with the findings by Shuval and Mizrach,^70^ as well as Shange,^71^ who emphasise BHPs’ inclination to control THPs and excluding THPs in collaborations within health settings. While the usefulness of having collaborating practitioners co-located cannot be denied, it is important to observe that unless THPs are included at senior decision-making level in these spaces, THPs become employees with little say on patient care. This could lead to dissatisfaction and less engagement by THPs in the plans towards collaboration. Therefore, discussions on power imbalances are important in coordinating collaboration.^71^

Although collaborations were sometimes enabled by systematic changes, such as political and government support, their organisation, cross-referrals were ad hoc and the client was the one navigating these referrals on their own. The role of the client in collaboration processes is something that is barely discussed in the literature. Kaptchuk and Miller^72^ state that having a client being mostly responsible for coordinating different treatments indicates weakness in the administration processes and the relationship between the practitioners, which are key ingredients in collaborations. A study by Ampomah^39^ concluded that a proper coordination of cross-referrals is a priority in enabling collaboration between the two practitioners. Guidelines and policies on collaborations should provide templates that practitioners can use while attempting collaborations.^39^

This study shows that the work of collaboration is complex and nonlinear. The substance of collaboration seemed to be linked with the time taken in investing in policies, resources and structures. It was noticed that attempts to collaborate are being made in LMICs, with 6 of the 10 studies coming from these counties. Furthermore, collaboration programmes are small scale and located in rural areas, where health resources are usually limited. This speaks to the recognition of THPs in expanding healthcare service delivery in areas where poor resources for health are reported. This is not to say these collaborations were out of challenges, but the support for them to occur ensured continuity even in the face of problems. As we move towards plans for universal health coverage and person-centred care, the need for this investment is crucial.

## Strengths and limitations

We believe that this is probably one of the first studies to summarise the evidence of existing collaboration attempts between THPs practitioners and BHPs, globally for all health conditions. We applied an extensive search method in seven databases, which also included searching for grey literature to insure we include the most relevant studies. The search period was also widened to map the literature published since 1978–2023, August. We notice that using only three databases in the latter two searcher is a possible limitation for this study. The reasons for this is because during initial search we found that of the eight databases, three sources yielded more results, and as such we were guaranteed to find the relevant literature.

We used the Rayyan software for screening data because this software allows for ‘blind’ screening, and we were able to minimise bias. This also increased the credibility of our screening process. It is also possible that searches in this topic existed under different terminologies that were not documented in the review. However, we included MeSH terms to help address this.

## Conclusion

From the studies reviewed, we were able to understand the structures that need to be in place to enable bidirectional collaborations to occur, the processes that take place within these collaboration programmes, as well as the factors that may hinder sustainability or scale up of these programmes. While the scoping approach does not allow for in-depth and detailed analysis of each of the collaboration cases presented here, it did map key aspects of these collaborations. A qualitative evidence synthesis, alongside primary studies that would provide an analysis of each programme, focusing on its mechanisms, collaboration influencers, processes and outcomes, is needed. Furthermore, capturing the experiences of the practitioners within those collaborations would be useful, particularly for programme implementers looking into replicating already existing collaboration programmes. Using case study methods in reporting these collaboration programmes will allow for better insights into the contextual components and dynamics of these collaboration programmes. This study confirmed that practitioner-level collaborations are few and their dynamics are similar in various settings, in that they depend on the formulation of dialogues and relationships, the practice of cross-referrals, opportunities for joint training, and treatment plan meetings. If countries are to achieve the integration of the two systems as per the WHO’s goal, more work is needed to move policy into implementation. Investing time in researching interpersonal, contextual and administrative hurdles to initiating and maintaining collaboration is one way to move policy plans into implementation.

## Appendix 1

**TABLE 1-A1:** Differences between protocol and final review.

Protocol	Final review
We intended to collect evidence of collaboration models between THPs and BHPs.	We decided to focus on collaboration attempts, rather than models, given the little programme development in this area.
We planned to use collect data up to 2020.	We extended the search period to 2023, to accommodate emerging literature in this area.
We planned to include data reporting all forms of what is regarded as collaboration in the literature, including task-shifting and consultations.	Upon refining our definition of collaboration, we decided to only focus on study that reports reciprocal or bidirectional collaboration.

## Appendix 2

**TABLE 1-A2:** Population, concept and context (PCC) framework and eligibility criteria for inclusion and exclusion of studies.

Criteria	Determinants	Inclusion criteria	Exclusion criteria
Population	**THPs**: THPs vary across countries and tribes	Articles reporting on collaboration with THPs who are classified as traditional healers, diviners and herbalists, alternative healers, native healers, aboriginal healers, indigenous healers, traditional Chinese healers, traditional Indian healers and Shammas	Articles presenting evidence on collaboration with faith healers, traditional surgeons, traditional birth attendants, midwives and doulas
	**BHPs:** All relevant BHPs	Articles reporting on different cadres of BHPs such as biomedical healers, general practitioner, psychologists, psychiatrists, medical doctors, nurses, medical doctors, physicians, healthcare workers and social workers	
	All participants are older than 18 years		
Content	**Collaboration practices** such as task-shifting, consultation, cooperation and integration of services	Evidence presenting one or more forms of collaboration at all levels of the continuum of care	Articles and studies that do not include collaboration between practitioners. Articles that report perceptions of collaboration
	**Medical conditions** such as mental health, HIV and AIDS, TB, cancer, and mild or chronic illnesses	Studies focusing on all health conditions	Studies focusing on animals
Context	**Global**	Articles published between January 1978 and November 2022 reporting global evidence	Evidence published in non-English languages
Sources of evidence		Evidence from empirical literature and grey literature including government documents, NGO reports, and academic dissertations. All study designs are considered.	Literature reviews, protocols, editorials, commentaries and news reports

## Appendix 3

**TABLE 1-A3:** The search strategy.

Key search words	(Traditional health practitioners[Text Word]) OR (Traditional healers[Text Word]) OR (‘aboriginal healers’[Text Word]) OR (alternative healers [Text word]) OR (‘indigenous healers’[Text Word]) OR (diviners[Text Word]) OR (herbalists[Text Word]) OR (traditional Chinese healers[Text Word]) OR (Traditional indian healers[Text Word]) OR (shammas[Text Word]) AND (biomedical health practitioners[Text Word]) OR (biomedical healers[Text Word]) OR (psychologists[MeSH Terms]) OR (psychiatrists[MeSH Terms]) OR (‘medical doctors’[Text Word]) OR (nurse[Text Word]) OR (‘social workers’[Text Word]) OR (‘healthcare workers’[Text Word]) OR (doctor[Text Word]) OR (physician[Text Word]) OR (‘medical staff’[Text Word]) OR (‘general practitioner’[Text Word]) AND (collaboration[Text Word]) OR (‘task shifting’[Text Word]) OR (integration[Text Word]) OR (cooperation[Text Word]) NOT (birth attendants, traditional[MeSH Terms]) NOT (midwives[MeSH Terms]) NOT (doulas[MeSH Terms]) NOT (‘traditional surgeons’[Text Word]) Filters applied: Journal Article, English, Complementary Medicine, from 1978/1/1† – 2020/3/30 *date updated to 2023/08/11*

† , The search start date was chosen because the recommendation for THP-BHP collaboration was officially supported in the WHO in the Alma Ata Declaration in 1978.
